# Culture of Human Embryos at High and Low Oxygen Levels

**DOI:** 10.3390/jcm13082222

**Published:** 2024-04-11

**Authors:** Ourania Konstantogianni, Theodoros Panou, Athanasios Zikopoulos, Charikleia Skentou, Sofoklis Stavros, Byron Asimakopoulos

**Affiliations:** 1 Laboratory of Reproductive Physiology-IVF, Faculty of Medicine, Democritus University of Thrace, 68100 Alexandroupolis, Greecetheopano2@med.duth.gr (T.P.); 2 Royal Devon and Exeter Hospital, Barrack Rd, Exeter EX2 5DW, UK; thanzik92@gmail.com; 3 Department of Obstetrics and Gynecology, Medical School of Ioannina, University General Hospital, 45110 Ioannina, Greece; 4 Third Department of Obstetrics and Gynecology, Attikon Hospital, Medical School, National and Kapodistrian University of Athens, 12462 Athens, Greece

**Keywords:** in vitro embryo culture, oxygen tension, embryo development, ROS, IVF, ICSI

## Abstract

One of the parameters potentially affecting the in vitro growth of preimplantation embryos is the oxygen concentration in the culture environment. An increased oxygen concentration causes the generation of ROS which in turn can cause damage to the cells and seriously disrupt the embryonic development. Previous studies have assessed oxygen concentrations in the fallopian tubes of several mammals of between 5 and 8%, while the oxygen levels in the uterus were found to be even lower; similar measurements have been confirmed in humans. In addition, studies in mammalian embryos showed that low oxygen concentrations improve embryo development. Multiple studies on the effect of the oxygen concentration on human embryos have been conducted so far with diverse methodologies and objectives. Data from these have been included in three meta-analyses. All meta-analyses indicate the potential benefit in favor of a low oxygen concentration, though data are considered to be of a low methodological quality and further studies are considered necessary. However, based on the existing evidence, it is suggested that a low oxygen concentration should be adopted in the routine of the IVF laboratory, especially in the case of blastocyst culture.

## 1. Introduction

In the first steps of in vitro fertilization (IVF), Steptoe et al. proposed the adoption of the three gases system (5% CO_2_/5% O_2_/90% N_2_) as suitable for the human blastocyst culture [1,2]. The beneficial effect of a low oxygen concentration on embryo culture has also been confirmed in animal models and particularly in in vitro mouse embryo cultures. The blastocyst development and quality of mice embryos was compared under three different oxygen concentrations (5, 20, and 40%). It was shown that mouse blastocysts cultured under 5% consisted of more blastomeres and numerically exceeded those developed under higher oxygen levels [3].

However, satisfactory results in the embryo culture under 20% O_2_ led to the use of N_2_ being abandoned in most IVF laboratories due to additional costs [2,3]. In the 1990s, researchers focused on the use of low oxygen in a human embryo culture due to the extension of the culture to the blastocyst stage which aimed to transfer fewer embryos in order to reduce multiple pregnancies [4]. The concept behind the increasing interest in applying lower levels of oxygen lies in the potential dangers associated with atmospheric oxygen concentrations. Several studies confirmed the harmful effect of reactive oxygen species (ROS) derived from the high oxygen levels present in the atmosphere [5,6]. The aim of the current review is to address the benefit of low oxygen levels on mammalian embryo development with particular emphasis on the human embryo development in assisted reproductive technology (ART). It also provides an insight into the physiological shifts induced by the different oxygen levels.

## 2. Oxygen Concentration in Female Reproductive Tract

There have been repetitive attempts to determine the physiological oxygen levels in the female reproductive tract in several mammals. In a study focusing on oxygen levels in monkeys, hamsters and rabbits, considerable differences were assessed: the oxygen levels varied from 1.5% in the monkey uterus to 8.7% in the rabbit fallopian tube and uterus and hamster uterus [7]. Similar results were presented for the oxygen levels in the rabbit oviduct: the assessed range varied between 40 mmHg (5.3%) and 75 mmHg (9.9%) [8]. A consensus range for the oxygen concentration in the female reproductive tract is 2 to 8% [2,9].

Other experimental studies provided an insight into the effect of different parameters on intrauterine oxygen tension in rats, hamsters and guinea pigs [10,11,12,13]. The impact of the menstrual cycle’s regulatory hormones (estrogens and progesterones) is of particular importance: the peak of the O_2_ levels was found under the effect of low estrogen and high progesterone levels [9,11]. It is noteworthy that oxygen levels are dependent on multiple physiologic (e.g., hormones, uterine cavity anatomy) and pathologic (e.g., cancer, infections) conditions and factors [9,14].

The studies conducted on humans are relatively few. Three major studies assessed the oxygen levels in the human female reproductive tract. The first, by Yedwab et al., compared the oxygen partial pressure among other parameters in women and female rats’ uteri throughout the menstrual cycle and observed an increase in oxygen levels by 86–90% in the ovulatory phase in humans (11 mmHg) and by 224% during the late proestrus phase in rats [15]. A more recent study observed a median oxygen pressure of 18.9 mmHg, corresponding to 11.8% oxygen air saturation with a wide range between 4 and 27% assessed. Pregnant women participating in the study exhibited lower levels at 9% oxygen air saturation, whereas non-pregnant women showed oxygen levels of 12.4% [16]. The last study documented uterine oxygen levels in women suffering from three types of gynecological malignancies and healthy controls. The pO_2_ levels were 28.5 mmHg for healthy controls and slightly decreased in all individuals suffering from cancer [14]. Overall, the oxygen concentration in the human uterine cavity has been assessed at almost 2%, ranging from 1.4 to 3.8% [14,15,16]. However, all three studies focused on different objectives and utilized different methods for the measurement of the oxygen levels.

Other studies attempted to assess the use of oxygen by the oocytes and the oxygen levels in the follicular fluid. In a study examining the composition of the human follicular fluid, Shalgi et al. observed the considerable variability of the oxygen levels in the 36 samples assessed. A mean oxygen tension of 54.3 mmHg was reported, corresponding to oxygen levels of almost 7% [17]. The exact way of oxygen utilization by the cumulus–oocyte complex was the objective of a mathematical model: it was concluded that dissolved oxygen is primarily consumed by the cumulus cells and scarcely reaches the oocyte, even under favoring conditions [18]. Contradictory results were the outcome of a different mathematical model undertaken on bovine and murine cumulus–oocyte complexes. It was found that cumulus cells consume only 0.25 to 0.5% of the total oxygen levels available and thus allow the oocyte to absorb most of it [19].

## 3. The Effect of Oxygen Levels on Early Embryo Metabolism

Throughout the preimplantation period the embryo depends on oxidative phosphorylation. During the cleavage state, the embryo metabolism relies primarily on pyruvate. The same metabolic pattern is evident in the morula stage as well. This substrate utilization analysis was performed in a culture microdroplet using an ultramicrofluorescence assay [20,21,22]. After compaction (blastomeres become increasingly coherent) and the blastocyst formation, a major metabolic shift occurs. The embryo metabolism becomes increasingly dependent on oxygen. Consequently, the embryo is capable of blastocoele formation and protein synthesis [23,24]. After the blastocoele formation, the blastocyst is comprised of the inner cell mass and the trophectoderm. These cells exhibit different energy requirements, as a mouse study verified. Trophectoderm cells use increasingly specific amino-acids, contain more mitochondria in comparison to the inner cell mass, produce more substrates and energy-containing substances (e.g., ATP) and thus consume greater amounts of oxygen [25].

ROS may result from either cellular metabolic reactions or the surrounding environment of the embryo. The main ROS produced from intracellular procedures include superoxide, hydrogen peroxide and hydroxyl radical [26]. The embryo protects itself from internal and external ROS by the use of specific antioxidant enzymes, including superoxide dismutase, glutathione peroxidase and gamma-glutamylcysteine synthetase [26]. External protective barriers are also present in the follicular and fallopian fluid: hypotaurine, taurine and ascorbic acid [27]. The diverse oxygen levels in the different parts of the female reproductive tract are also strongly associated with the increasing vulnerability due to potential ROS formation. The gradients of oxygen levels throughout the fallopian tubes and the uterus seem to play a stronger protective role for the early-stage embryo than several antioxidant enzymes [24].

The embryos are confronted with excessive amounts of ROS in an ART laboratory setting. The arrest of embryos’ development or the presentation of any other developmental disturbance has been accompanied by increased ROS in vitro, but not in vivo [28]. Consequently, ROS from external sources pose an important threat to normal development and pregnancy outcomes. Potential sources of ROS include increased oxygen levels, light or even the sperm cells necessary for fertilization. The potential harmful effect of increased oxygen levels has been confirmed from mouse models. A product of superoxide was targeted using fluorescence. It was demonstrated that mice cultured under lower oxygen levels (under 5% O_2_) exhibited the lowest fluorescent emissions compared to higher oxygen levels (20% and 40%) and the effect was dose-dependent [29]. In a similar staining-based study, the peroxide levels were found to be significantly decreased both in the two-cell and four-cell stages of embryo cultures under 5% O_2_ [30]. In another study investigating the detrimental impact of light exposure on hamster embryos, it was found that the generation of H_2_O_2_ during light exposure was significantly greater when the embryos were cultured at 20% O_2_ rather than 5% [31].

The direct effect of the ROS on the embryo metabolism of carbohydrates, lipids and amino acids has been studied extensively. Some data are also based on animal models, mostly mice [32,33]. Recent studies have focused on a more comprehensive analysis of the ROS’ impact on proteomics and even epigenetics, through the impact of dioxygenases and the paternal protamines on histone modification [34,35,36]. In a time-of-flight (TOF) spectrometry analysis of five preimplantation mice embryos, 32 proteins were found which could serve as potential biomarkers. Mice cultures under 5% O_2_ exhibited a significant resemblance to in vivo developed embryos, whereas the analysis of mice cultures under 20% oxygen showed the decreased expression of 10 proteins [37]. The gene expression patterns are also affected by the oxidative stress. Rinaudo et al. observed that embryos cultured under 5% O_2_ showed gene expression patterns that are expected in embryos developed in vivo [38]. The outcome of this study corresponds to the outcome of the study focusing on proteomics. Both of them imply that low oxygen tension creates similar conditions for the embryo’s development in vivo. The well-established toxic impact of ROS can be restricted, particularly in the IVF setting. Researchers have proposed potential ways in that direction. Apart from including substances with anti-oxidant actions in the culture medium, restricting light exposure to the culture media and the embryo culture oil and restricting the duration of gamete coincubation in order to avoid ROS production from the sperm metabolism, researchers have particularly emphasized one other method. This method uses lower oxygen tensions in the ART laboratory during insemination, fertilization and other relative procedures [6,39].

## 4. Culture of Mammalian Embryos under Low Oxygen Levels

The importance of the oxygen levels in embryo cultures is well-established. Since 1978, multiple studies have been conducted on various animal models, demonstrating the need for lowering oxygen levels. The decreased oxygen tension has resulted in better results in all studies carried out so far in various animal species [39].

Most of the animal-model-based studies exploring the benefits of a lower oxygen concentration on embryo cultures were conducted on rodents, including mice, rats and hamsters (Table 1). The first one, by Quinn et al., which took place back in 1978, focused on the oxygen levels’ impact on preimplantation embryo culture parameters and subsequent embryo quality. The study examined different oxygen levels: 5% O_2_ (classified as low) and 20/40% O_2_ (classified as high). It concluded that a culture under 5% O_2_ was beneficial for the preimplantation embryos, as this condition allowed more embryos to progress to the blastocyst stage and resulted in better quality embryos with an increased number of blastomeres [3]. The authors also provided an insight into a potential physiological mechanism behind the decreased blastomere number in embryos cultured under high oxygen levels: the blastomeres may perceive the oxygen levels as a marker for their position in the embryo, from the core to the periphery. Under a high oxygen level, they tend to misunderstand their position and thus divide at a slower rate [3]. Another study attempted to clarify the exact stages of embryo development which are affected by different oxygen concentrations. Therefore, they allocated the examined embryos, at first, in two culture groups (under 5% O_2_ or 20% O_2_) for the first 2 days of culture and afterwards they randomized the embryos for the next 2 days under the two possible oxygen levels, which resulted in four groups. They concluded that the oxygen levels affect the outcome both during the cleavage stage and after the compaction stage. The decrease in the oxygen levels after 48 h was not linked to a substantial improvement of the parameters examined. Likewise, all embryos which were cultured for a certain time interval under high oxygen levels had to withstand damage, which was not reimbursed after the oxygen levels’ shift. An additional outcome of the study is the fact that cleavage delay was particularly high in embryos cultured under 20% O_2_ [40]. Two other studies with mice primarily examined the genetic status and the way it may be affected by different oxygen levels. In a FISH-based study, BALB/cWT mice, which are susceptible to Y chromosome abnormalities, were examined. Increased mosaicism was observed among embryos cultured under 20% O_2_, whereas embryos cultured under 5% oxygen had mosaicism levels comparable to the control branch of the study [41]. Apart from the effect on the gene expression pattern analyzed previously, the microarray study by Rinaudo et al. verified that a culture under 5% oxygen leads to an increased rate of embryo development and an increased cell number [38]. Kishi et al. [42] showed that hamster embryos cultured in hamster embryo culture (HEMC-1) had a higher blastocyst rate under 5% O_2_ than under 20% O_2_ levels (20.1% vs. 5.5%, respectively). In another study, where multiple parameters were evaluated, it was found that the progression of hamster 2-cell embryos to the blastocyst stage was facilitated under 5% or 10% oxygen levels, whereas 10% O_2_ was identified as the optimal oxygen level for hamster embryo cultures [43].

The blastocyst rate and the related parameters were in the epicenter of a rabbit embryo study, carried out under multiple oxygen levels in two groups (5, 10 and 15% O_2_ and 1, 5 and 20% O_2_). In the first group, cultures under 5, 10 and 15% O_2_ resulted in 48, 38 and 21% of the embryos reaching the hatching blastocyst stage, with 258, 226 and 188 cells per embryo, respectively. In the second group, cultures under 1, 5 and 20% O_2_ led to a 67, 72 and 29% proportion of hatching embryos, respectively. For the same group, the average cells per embryo were significantly higher under 1% O_2_ and 5% O_2_ compared to 20% O_2_ [44]. The study by Karja et al. examined the effect of different oxygen levels (8–10% and 20%) on the in vitro maturation (IVM) of porcine oocytes, IVF and in vitro embryo production. It was shown that a similar number of oocytes reached the MII phase and were fertilized after IVM. The beneficial character of the low oxygen levels (8–10%) was reflected in the blastocyst formation rates. In addition, DNA fragmentation was found significantly lower under low oxygen levels (8–10%) [45]. Goat two- to four-cell embryos were cultured for a 6-day interval either under 20% O_2_ or 7% O_2_ in another comparative study. In total, 80% of the embryos reached the expanded or/and hatched blastocyst stage under 7% O_2_ in contrast to merely 29% in the other group. Also, the mean embryo cell number was higher under the low oxygen levels’ culture (7%) [46]. Leoni et al. examined bovine embryo cultures under 5% and 20% oxygen levels. The blastocyst formation rate, both on the 6th and on the 7th day under 5% O_2,_ emerged as significantly higher (63.04% vs. 47.36% and 35.10% vs. 26.09%, respectively) [47]. A more recent study focused on the oocyte maturation and embryo culture of the yak species under different oxygen levels (20% O_2_, 10% O_2_, 5% O_2_, 1% O_2_) [48]. This study also confirmed that oocyte maturation, blastocyst and hatched blastocyst rates as well as the total blastocyst cell number, the inner cell mass cells and the trophectoderm cells were significantly higher under 5% O_2_. The study also addressed the effect of ultra-low oxygen levels: the oocyte maturation, the cleavage, the blastocyst and the hatched blastocyst rates under 1% O_2_ were the lowest assessed [48]. In the last two studies mentioned, the cleavage rates were found to be significantly higher under high oxygen levels [47,48] and it was speculated that the detrimental effects of high oxygen levels are mainly manifested on day 3 with blastulation, more than in the earlier development [48]. The latter study also analyzed other molecular parameters, including the apoptosis index of oocytes and blastocyst cells and the expression patterns of genes related to metabolism, antioxidant response, apoptosis, oocyte competence and embryonic developmental markers. All of them were the lowest under 5% O_2_ and the highest under 1% O_2_ [48]. We could speculate that the lower outcome with 1% O_2_ indicates that this extra-low oxygen level is inadequate to support embryo metabolism.

A recent study investigating the effects of oxygen levels on IVM, IVF and the embryo development of common marmosets (*Callithrix jacchus*) gave interesting results which underline the species-specific differences in these processes [49]. IVM and IVF were performed under 8% or 20% O_2_ with the latter giving better results, whereas the embryo culture was performed under 5% or 20% O_2_, with 5% O_2_ resulting in a better embryo morphology and developmental rates [49]. The authors speculated that during IVM and IVF procedures, the cumulus cells surrounding the oocyte act as a barrier which lowers the amount of O_2_ available for the oocyte and, at the same time, protects it from excessive oxidation; hence, with 20% O_2_, the oocyte has acceptable O_2_ tension, whereas with 8% O_2,_ it is probably in hypoxic conditions. They also suggested that the use of oil covering the microdroplets of the culture medium which hosts the oocytes (during IVM and IVF) and the sperm (during IVF) is also another factor lowering the O_2_ tension [49].

It is obvious that the above mentioned animal studies, summarized in Table 1, provide sufficient evidence of the culture in οxygen levels lower than 20% for embryo development. Although the optimal level of oxygen differs by the species, most of the studies found that culture under 5% O_2_ is beneficial.

**Table 1:** Summary of major animal studies KSOM, potassium-supplemented simplex optimized medium; IVM, in vitro maturation; IVF, in vitro fertilization; HECM-1, hamster embryo culture medium-1; Mkrb, modified Krebs–Ringer bicarbonate solution; SOF, synthetic oviduct fluid; TUNEL, terminal deoxynucleotidyl transferase-mediated deoxyuridine triphosphate-nick end labeling; WT, wild type; BSA, bovine serum albumin; FISH, fluorescence in situ hybridization.

Study	Species	Objective	Primary Outcomes	Secondary Outcomes and Additional Remarks	Reproductive Method
Rinaudo et al. (2006) [38]	Mouse	Effect of oxygen levels (5% or 20%) on gene expression patterns	- 5% O_2_ levels were correlated to increased rate of embryo development and increased cell number. - The gene expression pattern under 5% O_2_ were similar to those present under in vivo development.	- Embryo culture in Whitten’s medium or KSOM medium with amino acids under 20% O_2_ or 5% O_2_. - Results assessed with the use of microarrays.	Not specified
Quinn et al. (1978) [3]	Mouse	Effect of different oxygen levels on preimplantation embryo culture	- More embryos progressed to the blastocyst stage under 5% O_2_. - Embryos under 5% O_2_ showed an increased number of blastomeres. - Increased time led to fewer blastomeres present.	- Optimal oxygen levels for embryo development were assessed between 2.5% and 5% O_2_. - Preimplantation embryo development took place under 5% O_2_ and 20/40% O_2_.	Not specified
Kishi et al. (1991) [42]	Rat	Effect of HECM-1 medium use on rat 1-cell embryo culture	- Under HECM-1, 57.9%, 32.2%, 17.4% and 9.9% of the pronuclear embryos progressed to the 4-cell, 8-cell, morula and blastocyst stage. - Under mKRB, pronuclear embryos barely reached the 2- and 4-cell stage and never progressed to stages beneath it. - The blastocyst formation rate was assessed at 20.1% under 5% oxygen levels and was significantly higher compared to rate of 5.5% under 20% oxygen levels.	- Embryos were cultured either under hamster embryo culture medium-1 (HECM-1) related to lower oxygen levels or modified Krebs–Ringer bicarbonate solution (mKRB).	Not specified
McKiernan et al. (1990) [43]	Hamster	Effect of different parameters on hamster embryo culture	- Progression of hamster 2-cell embryos to the blastocyst stage was facilitated under reduced oxygen levels. - 10% O_2_ among other conditions identified as optimal setting for hamster embryo culture, as 51–57% of the hamster 2-cell embryos progressed to the blastocyst stage.	- Temperature under 37.5 °C emerged as inhibitory for the culture. - Increased CO_2_ (7.5% and 10%) supportive for hamster embryo culture. - No significant effect of electrolytes and other chemical substances observed. - Silicone overlay helpful in the culture medium. - Osmolarity above 325 m. Osmols inhibited culture progression.	Not specified
Li et al. (1993) [44]	Rabbit	Effect of different oxygen levels (1–20% O_2_) on progression to blastocyst and blastocyst-related parameters	- In the first group, culture under 5, 10 and 15% O_2_ resulted in 48, 38 and 21% of the embryos reaching the hatching blastocyst stage with 258, 226 and 188 cells per blastocyst, respectively. - In the second group, culture under 1, 5 and 20% O_2_ led to 67, 72 and 29% proportion of hatching embryos, respectively. - In the second group, average cells per embryo were significantly higher under 1% O_2_ and 5% O_2_ compared to 20% O_2_.	- 2 study groups with culture under different oxygen levels (5, 10 and 15% O_2_ and 1, 5 and 20% O_2_).	Not specified
Karja et al. (2004) [45]	Pig	Effect of different oxygen levels (8–10% or 20%) on oocyte characteristics and embryo quality parameters	- Similar numbers of oocytes reached MII phase and were fertilized. - Progression to blastocyst rates and mean blastocyst cell rates were significantly higher under lower oxygen levels (8–10%). - Significantly lower DNA fragmentation levels in blastocysts under lower oxygen levels (8–10%) were observed.	- Comparison between a standard and a portable incubator. - Comparison between culture under 8–10% O_2_ and 20% O_2_. - DNA fragmentation assessment in the blastocyst with TUNEL.	IVM/IVF/in vitro culture
Batt et al. (1991) [46]	Goat	Effect of different oxygen levels (7% or 20%) and different protein sources in the culture medium on preimplantation embryo parameters	- Significantly higher number of embryos (80% vs. 29%) reached the expanded or/and hatched blastocyst stage under 7% O_2_. - The mean number of cells emerged higher in embryos under 7% O_2_.	- 2- to 4-cell goat embryos were examined and cultured in synthetic oviduct fluid (SOF) for a 6day interval under 20% O_2_ or 7% O_2_. - Miles bovine serum albumin and human serum were the 2 protein sources with the best features in relation to the mean number of embryo cells.	Not specified
Leoni et al. (2007) [47]	Ovine	Effect of oxygen levels (5% or 20%) on ovine embryo culture quality parameters	- Blastocyst formation on the 6th day under 5% O_2_ emerged significantly higher (63.04% vs. 47.36%). - Blastocyst formation on the 7th day under 5% O_2_ emerged significantly higher (35.10% vs. 26.09%). - Viability rates post vitrification, total cell number and the inner cell mass cells to trophoblastic cells ratio were similar between the cultures under the 2 oxygen levels on 3 consecutive days (6th, 7th and 8th day).	- Fertilization took place either under 5% O_2_ or under 20% O_2_ and culture under SOF + aa + 0.4% BSA in 5%CO_2_ and 5% O_2_. - Significantly higher cleavage rate was observed under 20% O_2_ (61.2% vs. 50.8%). - No differences in assessed cleavage distribution at 22 h, 26 h and 40 h	IVF
Bean et al. (2002) [41]	Mouse	Effect of oxygen levels (5% or 20%) on genetic status	- Increased mosaicism was observed under 5% CO_2_. - Control-level mosaicism was observed under 5% O_2_.	- BALB/c WT male mice examined with increased tendency to Y chromosome abnormalities. - FISH was used for the assessment of the sex chromosomes during different cell stages (2-, 4-, 8- and 16-cell stage).	IVF
Wale et al. (2010) [40]	Mouse	Effect of oxygen levels and oxygen level shifts on preimplantation embryo culture	- Cleavage delay was present for embryo culture under 20% O_2_ compared to 5% O_2_. - The oxygen level shift from 20% O_2_ to 5% O_2_ after 48 h did not improve culture-related parameters substantially. - All embryo cultures under 20% O_2_ for a time period showed significantly lower blastocyst cell numbers. - Oxygen levels affect embryo culture during the cleavage stage and after the compaction stage.	- Mice were cultured under 5% O_2_ or 20% O_2_ during the first 2 days and then they were randomized in four groups for other 2 days. - Embryo imaging every 15 min took place under time-lapse microscopy.	Not specified
He et al. (2020) [48]	Yak	Effect of different oxygen levels (20% O_2_, 10% O_2_, 5% O_2_ or 1% O_2_) on oocyte maturation, embryo preimplantation potential and other parameters	- Oocyte, blastocyst and hatched blastocyst maturation rates emerged as significantly higher under 5% O_2_. - Cleavage rate under 10% O_2_ and 20% O_2_ were significantly higher compared to the 5% O_2_ group. - Oocyte, blastocyst and hatched blastocyst maturation rates and the cleavage rate under 1% O_2_ were the lowest assessed. - Total blastocyst cell number, the inner cell mass cells and the trophectoderm cells were significantly higher under 5% O_2_.	- The apoptosis index of oocytes and blastocyst cells was the lowest under 5% O2 and the highest under 1% O_2_. - Expression patterns of genes related to metabolism, antioxidant response, apoptosis, - Oocyte competence and embryonic developmental markers were significantly different under 5% O_2_.	IVF
Tkachenko et al. (2017) [49]	Marmoset	Effect of paired oxygen levels (8% O_2_–5% O_2_, 20% O_2_–20% O_2_, 20% O_2_–5% O_2_ and 8% O_2_–20% O_2_) on in vitro maturation (IVM) and in vitro fertilization (IVF), respectively.	- Oocyte maturation rate emerges as significantly higher under 20% O_2_ in comparison to 8% O_2_. - Embryo-related morphology parameters and progression rates (from 4-cell embryos to morula stage) showed a comparative benefit for the 5% O_2_ group in IVF over the 20% O_2_ group.	- No significant difference between cumulus expansion in IVM under 20% O_2_ or 8% O_2_ was observed. - Progression to the second metaphasis stage was significantly better in the 20–20% group compared to the 8% O_2_–5% O_2_ and 20% O_2_–5% O_2_ groups. - Fertilization was impossible for the 8% O_2_–20% O_2_ group. - The highest normal spindle rate was assessed in the 20–20% group and the difference was statistically significant. - A significantly higher proportion of fertilized oocytes achieved first cleavage in the 20–20% group.	IVM/IVF

## 5. Culture of Human Embryos under Low Oxygen Levels

Although Steptoe et al. proposed in their monumental study that the human embryo culture should take place under low oxygen levels, the atmospheric oxygen levels dominated for years in the ART laboratories [1]. Some researchers attempted to explain this trend and concluded the following possible explanations: the need for a N_2_ supply and new incubators that entail additional costs, the improvement of culture media used in human IVF procedures and the embryo transfer in the four-cell or eight-cell stage on the 2nd or the 3rd day, respectively [4]. However, the culture of human embryos under low (5%) oxygen levels is gaining ground and a lot of studies seem to support the adoption of this method in everyday laboratory setting.

Overall, the results of twelve prospective randomized studies, two randomized studies, four prospective randomized controlled trials, four randomized controlled studies, three comparative studies, one retrospective study, one monocentric retrospective observational study, one cohort study, one retrospective cross-sectional study, one meta-analysis, one review with quantitative synthesis and one systematic review with meta-analysis are presented (Table 2).

All of the studies addressed the impact of low oxygen levels on human embryo cultures and their related parameters. The number of participants in the studies varied significantly, ranging from 100 oocyte donation receivers to 1382 patients. Some studies did not specify the exact number of patients or participants and referred merely to the total number of IVF cycles or the oocyte retrieval cycles undertaken. It is of note that both fertilization methods were used, either IVF, ICSI or a combination of both.

In order to examine whether the human embryo culture under 5% oxygen is indeed advantageous, several parameters have been assessed. The most frequent parameters examined were the fertilization rate, criteria related to blastocyst and embryo quality, developmental rate, implantation and pregnancy rate. One of the first studies published by Dumoulin et al. indicated a significantly increased pregnancy rate under low oxygen levels (24.2% under 5% oxygen in comparison to 19.4% under atmospheric conditions) [50]. In another study by the same researchers, there was a significant improvement to the blastocyst formation rate (25.8% vs. 20.4%) and blastocyst quality under 5% oxygen [51]. The better embryo quality was evaluated as a common finding in many studies, also by de los Santos et al., who referred particularly to the increased blastomere number [52]. In another prospective randomized study simultaneously assessing both fertilization methods, only the embryo score on day 3 significantly improved in cultures under 5% oxygen, without affecting the fertilization rates [53]. Meintjes et al. identified strong proponents of the embryo culture under 5% oxygen, as they found both better preimplantation development (42.9% vs. 30.7%) and an increased living birth rate (57.4% vs. 42.6%) [54].

Sibling oocyte development was a striking feature of another prospective randomized study. The embryos cultured under 5% oxygen exhibited an increased blastocyst formation rate for both fertilization methods (IVF: 73.2% vs. 63.1% and ICSI: 67.4% vs. 54.7%), an increased blastocyst quality (IVF: 31.1% VS. 14.6% and ICSI: 18.9% vs. 11.4%) and a significantly increased optimal blastocyst formation ratio on day 5 (at 2.1 for IVF and 1.7 for ICSI). Although all the previously mentioned outcomes were clearly in favor of the culture under 5% oxygen, the fertilization rates were found to be unaffected with both fertilization methods (IVF: 59% vs. 43.2% and ICSI: 51.2% vs. 28.5%) [55].

Nanassy et al. conducted a retrospective study in order to examine the way in which the oxygen level shifts affected the embryo quality, pregnancy and implantation rate between the 3rd and the 5th day. Until the 3rd day, all embryo cultures took place under 20% O_2_. After the 3rd day, 189 and 193 patients were randomized for the group with cultures under 5% O_2_ and 20% O_2_, respectively. No significant difference could be observed in terms of the embryo quality, implantation rates (44.06% under the 5% O_2_ vs. 44.16% under 20% O_2_), the pregnancy rate (71.27% under the 5% O_2_ vs. 78.72% under 20% O_2_) and the clinical pregnancy rate (58.56% under the 5% O_2_ vs. 64.36% under 20% O_2_). This study confirmed the presumption put forward by another study with mice showing that cultures already damaged un-der high oxygen levels can not improve if the oxygen tension is reduced [40,56]. In another study by Peng et al., higher fertilization and implantation rates were observed primarily for the group of embryos cultured under low oxygen levels. However, this was the first study to indicate that embryos cultured under 20% O_2_ showed significantly higher fertilization rates, implantation rates and quality compared to embryos cultured under high oxygen levels for the first 2 days and then cultured under lower oxygen levels. This study speculated that embryos are more sensitive to oxygen level shifts than to oxygen levels per se [57].

The birthweight in embryos developed under low oxygen levels was assessed in the unique cohort study by Van Montfoort et al. The study could not link the oxygen levels to the birthweight. However, a significant increase in embryo quality was reported (45.8% under 5% O_2_ and 30.9% under 20% O_2_). Although there was no correlation to the live birth rate, embryos of good quality were eligible for cryopreservation [58]. A significant increase in cryopreservation eligibility for embryos cultured under low oxygen levels (30.8% under 5% O_2_ and 19.2% under 20% O_2_) was also observed by Sepulveda et al., together with better embryo quality (55.4% under 5% O_2_ and 41.8% under 20% O_2_). All the other parameters assessed did not reach statistical significance, i.e., implantation rates (41.8% under 5% O_2_ and 36.8% under 20% O_2_) and pregnancy rates (55.3% under 5% O_2_ and 54.6% under 20% O_2_) [59]. Also, Waldenstrom et al. observed a higher number of eligible blastocysts for cryopreservation (1.7 in the culture under 5% O_2_ vs. 1.1 in the culture under 19% O_2_) together with a satisfactory blastocyst formation rate (47.8% in the culture under 5% O_2_ vs. 42.1% in the culture under 19% O_2_) and the mean number of available blastocysts (3.8 in the culture under 5% O_2_ vs. 3.3 in the culture under 19% O_2_) [60]. In a prospective randomized study, Bahceci et al., found that although the embryo quality in the transfers on day 3 was better in cultures under 5% O_2_, the authors concluded that the oxygen levels did not particularly affect the outcome. It is of note that this study did not evaluate the embryos’ progression until the blastocyst stage [61]. The better quality of the embryos is a concept shared by another study [62] which reported optimal blastocyst features under 5% O_2_. However, the low oxygen levels did not show a significant impact neither on implantation rates (28.8% under 5% O_2_ and 25.2% under 20% O_2_) nor on ongoing pregnancy rates (31.6% under 5% O_2_ and 27.1% under 20% O_2_), although they were beneficial for the pregnancy rates in the special subgroup of poor responders (23% under 5% O_2_ and 9.8% under 20% O_2_) [62]. In contrast to the previous findings, Kasterstein et al. observed a significant increase both in the implantation and pregnancy rates with cultures under 5% O_2_ [63]. Only the fertilization rates were found to be relatively similar between the two groups assessed. An increased number of blastomeres and an optimal embryo quality were also parts of the findings, explaining also the significantly higher number of embryos eligible for transfer (31.6% under 5% O_2_ vs. 23.1% under 20% O_2_). In this study, the live birth rate was significantly higher under the low oxygen levels’ culture (34.2% under 5% O_2_ vs. 15.8% under 20% O_2_) [64]. Like all the previous studies, Ciray et al. emphasized the significantly better embryo and blastocyst quality observed [64].

Paternot et al., in a randomized controlled trial, evaluated the quality of embryos up to day 3 under 5% or 20% O_2_ without finding significant differences [65]. This is the only study that actively discourages from the use of low oxygen levels in embryo cultures [65].

Ruiz and her collaborators in IVI performed a prospective randomized controlled trial to evaluate the efficacy of group embryo cultures under 5% O_2_ in benchtop incubators, whereas the control group embryos were cultured individually at 20% O_2_ in common large incubators. In the statistical analysis, fresh embryo transfers as well as frozen ones were included [66]. With this strategy, the fertilization rate was not different, but there was a higher blastocyst rate, implantation and live birth rate with the fresh embryo transfers [66]. Taking into consideration the frozen embryo transfers, the cumulative implantation rate and cumulative live birth rate were higher as well [66].

Another team from Zagreb performed a prospective randomized trial comparing benchtop incubators with 5% O_2_ and conventional incubators with 20% O_2_ [67]. The only results in favor of the benchtop incubators with 5% O_2_ was the number of blastocysts on day 5 and clinical pregnancy after a single blastocyst transfer; however, there was not any improvement in the clinical pregnancy for all subgroups, nor in the live birth rate, which was the primary outcome of this study [67].

Although almost all the studies compare 5% O_2_ to 20% O_2_, a Chinese team investigated the effects of continuous embryo culture under ultra-low (2%) O_2_ tension vs. 5% O_2_ tension [68]. They did not find differences in the embryo development nor in the clinical or ongoing pregnancies between the two groups and, therefore, they concluded that the continuous culture under 2% O_2_ does not give any advantage for the in vitro development of human embryos [68]. Similarly, the team of M. Bedaiwy performed a study comparing 3.5% O_2_ to 5% O_2_. The results were disappointing for the cultures under 3.5% O_2_: though the fertilization and cleavage rates were better, the compaction rate, the number of high-quality blastocysts, the implantation rate and the clinical pregnancy rate were in favor of cultures under 5% O_2_ [69].

In a meta-analysis, no statistically remarkable difference was assessed between the cultures at 5–6% O_2_ or 20% O_2_ concerning fertilization, implantation and ongoing pregnancy. The only benefit observed under low oxygen levels referred to embryos transferred on day 5 and day 6. Nevertheless, the authors consider additional studies as advisable [70]. In contrast, a review with quantitative synthesis concluded that low oxygen (5–6%) levels were beneficial as living birth rates, clinical pregnancy rates and ongoing pregnancies were positively affected, leading to an increase in the success rates from 32% to 43%, without any increase in multiple pregnancies, miscarriages and congenital deformities [71]. In the most recent meta-analysis, Nastri et al. found no difference between 5 and 6% O_2_ and 20% O_2_ regarding fertilization and the cleavage rate, a better morphology at the cleavage stage with 5–6% O_2_ and they observed a small improvement in the live birth/ongoing pregnancy and clinical pregnancy rates with the use of 5–6% O_2_ [72]. However, they underlined that the available evidence is of very low quality; therefore, more large, well-contacted, randomized clinical trials are needed [72].

Nevertheless, new culture strategies appeared. Several researchers, taking into account the conditions recorded to prevail in the female reproductive system, proposed and tested a shift from 5% O_2_ to 2% O_2_ after day 2–3, i.e., at the morula and blastocyst stages. It is evident that the strategy of using two phases of low oxygen concentrations (5% in the first 2–3 days of preimplantation development and 2% thereafter) better simulates the physiological conditions to which embryos are exposed within the female reproductive system. However, the reported results are contradictory. In 2016, Yang et al. thawed day-3 embryos that had been cultured at 20% O_2_ and further cultured either at 2% O_2_, 5% O_2_ or 20% O_2_ [73]. There were no differences among the three groups in terms of the number of blastocysts and the number of high-quality blastocysts [73]. In the same year, Kaser et al., in a randomized controlled trial, used bipronucleate and tripronucleate embryos allocated either for continuous culture in 5% O_2_ or in 5% O_2_ from day 1 to day 3 and 2% O_2_ from day 3 to day 5 [74]. Although they found that the total yield of blastocysts was higher with the biphasic culture, these blastocysts had fewer cells than the blastocysts derived under 5% O_2_ [74]. De Munck et al. used the same biphasic oxygen concentration strategy without finding any improvement in embryo development, quality and the utilization rate of blastocysts [75]. On the contrary, Broillet et al., in an observational retrospective study, reported a significant improvement in the total and usable blastocyst rates as well as a higher cumulative birth rate with the biphasic (5–2%) oxygen concentration strategy [76]. Recently, Li et al. reported that the biphasic strategy (5–2% O_2_) resulted in a better blastulation rate for low-quality cleavage embryos, but not for high-quality cleavage embryos [77]. The most recent study is a retrospective cross-sectional one that found only marginal benefits with the biphasic (5–2%) oxygen strategy: they performed both fresh and frozen embryo transfers, finding that there was no difference in the pregnancy and implantation rates although, in fresh embryo transfers, the implantation rate of embryos cultured with the biphasic oxygen strategy was significantly better [78].

Recently, a new embryo culture strategy has been tested. Herbemont et al., based on the results of Wale and Gardner’s study in mouse embryos [40], designed and performed a prospective randomized study with multiple arms: culturing preimplantation human embryos initially with either 20% O_2_ or 5% O_2_ and then (day 5–6) with 20% O_2_ and either 5% O_2_ or 2% O_2_ [79]. The hypothesis for this study was that embryos are particularly susceptible to oxidative stress during the first 2 days of development, whereas after the activation of the embryonic genome, they can develop unhindered even in atmospheric oxygen conditions [79]. The primary study endpoints were the day-2 embryo quality and blastocyst quality. According to the results of this study, on day 2, higher division rates and more high-quality embryos were obtained in the 5% O_2_ culture than in the 20% O_2_ culture. Culturing blastocysts in either 5% O_2_ or 2% O_2_ gave better-quality blastocysts than the culture in 20% O_2_ [79]. However, there were no statistically significant differences in the clinical outcome of the blastocyst transfer, regardless of whether they were grown under atmospheric oxygen or hypoxic conditions [79]. Therefore, although it has been re-established that embryos are particularly vulnerable to 20% O_2_ conditions during the first 2 days of development, the use of low oxygen concentrations (2% or 5%) appears to be advantageous for obtaining good-quality blastocysts [79].

It is obvious that there are considerable differences between the aforementioned studies regarding the settings, the primary outcomes, the size of the study populations and not all of them are randomized clinical trials (Table 2). Therefore, it is not strange that the meta-analysis of Nastri et al. found that the available data are of a low quality [72]. On the other hand, it is clear that all but one of these studies found that cultures of human embryos under 5–6% O_2_ give some advantage over the cultures under atmospheric O_2_. Hence, we can draw some conclusions with considerable certainty: 1. The fertilization rate, especially with ICSI, seem to not be influenced significantly by the oxygen tension. 2. Embryo development is better under 5% O_2_ and this is more obvious in the blastocyst formation rate and quality. 3. In our opinion, an initial culture under 20% O_2_ and a shift to 5% O_2_ after 2–3 days does not guarantee any advantage. 4. The use of a biphasic oxygen strategy (5% during the first three days, 2% afterwards) is promising as it appears to better reproduce the physiological conditions; however, the existing evidence is not strong enough to support this.

**Table 2:** Summary of major human studies. IVF, conventional in vitro fertilization; ICSI, intracytoplasmic sperm injection; PNs, pronuclei.

Study	Study Type	Objective	Study Population	Outcome	Reproductive Method
Dumoulin et al. (1995) [50]	Prospective randomized study	Evaluation of oxygen levels (5% CO_2_/5% O_2_/90% N_2_ or 5% CO_2_/20% O_2_) on fertilization rates, development during transfer day, implantation and pregnancy rates	257 IVF cycles from 186 patients	- Clinically relevant pregnancy rates were assessed at 24.2% under 5% O_2_ in comparison to 19.4% under 20% O_2_ - No major difference in other parameters between the two groups.	IVF
Dumoulin et al. (1999) [51]	Prospective randomized study	Evaluation of oxygen levels (5% O_2_ or 20% O_2_) on fertilization rates, implantation rates and development during 2nd or 3rd day	1380 IVF cycles	- No major difference was evaluated in fertilization rate (60% in 5% O_2_ vs. 61% in 20% O_2_), implantation rates (13.4% vs. 14%) and development during 2nd or 3rd day (26.6% vs. 25.4%). - Statistically significant increased rates in blastocyst formation per cycle were in 5% O_2_ group assessed (25.8% vs. 20.4%). - Microscopic blastocyst classification was also higher in O_2_ group both for blastocysts fixed at day 5 and day 6 (smaller number of cells in 20% O_2_ group). - The authors consider the increase in preimplantation rate as inadequate to produce better pregnancy rates or they speculate that oxygen levels are crucial in the later stages of preimplantation.	IVF
Bahceci et al. (2005) [61]	Prospective randomized study	Comparison of embryo culture under 5% O_2_ or 20% O_2_ in relation to the ICSI outcome	822 oocyte retrieval cycles, 712 of which led to embryo transfer: - 255 transfers on 2nd day (118 cycles cultured under 5% O_2_ and 137 under 20% O_2_). - 457 transfers on 3rd day (219 under 5% O_2_ and 238 under 20% O_2_).	- Quality of embryos and clinical outcomes were similar between the two groups. - Embryos transferred on the 3rd day under 5% O_2_ exhibited better features. - ICSI cycles’ outcomes were not particularly affected by oxygen levels. - The development of the embryos until the blastocyst formation was not assessed.	ICSI
Kea et al. (2007) [53]	Prospective randomized study	Evaluation of the oxygen levels’ effect (5% CO_2_/5% O_2_/90% N_2_ or 5% CO_2_) on fertilization rates, embryo development and pregnancy outcomes in IVF patients	1045 oocyte retrievals from 106 patients: - 35 patients underwent IVF, 47 patients underwent ICSI and 2 followed a combined procedure on the 3rd embryo transfer day. - 8 patients underwent IVF, 12 patients underwent ICSI and 2 followed a combined procedure on the 5th embryo transfer day.	- No major differences were concluded in fertilization rates, patients’ age and fertilization profile, total number of oocytes and 2PNs and pregnancy rates. - The mean embryo score assessing embryo development was significantly higher on the 3rd day. - 21% of the participants were eligible for blastocyst transfer. - Embryo transfer was performed for 20% under 5% CO_2_/5% O_2_/90% N2 and 21% under 5% CO_2_ on the 5th day.	IVF/ICSI/IVF and ICSI (combination)
Kovacic et al. (2008) [55]	Prospective randomized study	Effect of different oxygen levels (5% O_2_ or 20% O_2_) on sibling oocyte development until the blastocyst stage and evaluation of fertilization rate and the ratio of optimal embryos and blastocysts	785 cumulus–oocyte complexes (COCs) in the IVF group: - 388 COCs under under 5% O_2_. - 397 COCs under 20% O_2_. 924 cumulus–oocyte complexes (COCs) in the ICSI group: - 462 COCs under under 5% O_2_. - 462 COCs under 20% O_2_.	- 5% oxygen levels resulted in increased rate of optimal embryos on the 3rd day (IVF: 59% vs. 43.2% and ICSI: 51.2% vs. 28.5%) without affecting fertilization rates either with IVF or ICSI. - Under 5% O_2_, the blastocyst formation rates were increased (IVF: 73.2% vs. 63.1% and ICSI: 67.4% vs. 54.7%) as well as the proportion of the blastocysts with normal inner cell mass (IVF: 31.1% VS. 14.6% and ICSI: 18.9% vs. 11.4%). - The optimal blastocyst formation ratio on the 5th day was assessed at 2.1 for IVF and 1.7 for ICSI, supporting the low (5%) oxygen levels.	IVF/ICSI
Ciray et al. (2009) [64]	Prospective randomized study	Evaluation of the oxygen levels’ effect (6% CO_2_/5% O_2_/89% N_2_ or 5% CO_2_/20% O_2_) on embryo qualities and blastocyst status	75 oocyte retrieval cycles including 2061 oocytes, 74 of which led to embryo transfer: - 869 oocytes for the control group. - 868 oocytes for the study group.	- Statistically significant improvements in embryo quality and in blastocyst status have been observed under 6% CO_2_/5% O_2_/89% N_2._ - It is of note that the shift from the control group (5% CO_2_/20% O_2_) to other group (6% CO_2_/5% O_2_/89% N_2_) occurred on the 3rd day.	ICSI
Meintjes et al. (2009) [54]	Prospective randomized study	Impact of low oxygen levels (5% O_2_) on pregnancy rates	230 patients undergoing IVF or ICSI: 115 control group (20% O_2_). 115 group (5% O_2_).	- Increased preimplantation rate with 5% O_2_ in comparison to 20% O_2_ levels (42.9% vs. 30.7%). - Increased living birth rates with 5% O_2_ in comparison to 20% O_2_ levels (57.4% vs. 42.6%).	IVF/ICSI
Waldenstrom et al. (2009) [60]	Prospective randomized study	Effect of different oxygen levels (5% O_2_ vs. 19% O_2_) on birth rate	396 patients: 197 patients for culture under 5% O_2._ 199 patients for culture under 19% O_2._	- The results showed a statistically significant increase in blastocyst formation (47.8% in the culture under 5% O_2_ vs. 42.1% in the culture under 19% O_2_), in the average blastocyst number (3.8 in the culture under 5% O_2_ vs. 3.3 in the culture under 19% O_2_) and in the average number of blastocysts for cryopreservation (1.7 in the culture under 5% O_2_ vs. 1.1 in the culture under 19% O_2_). - Blastocyst culture under low oxygen levels increased birth rate by 10%.	IVF
Nanassy et al. (2010) [56]	Retrospective study	Impact of oxygen levels’ shift (5% O_2_ and 20% O_2_) on embryo quality, implantation and pregnancy rate between the 3rd and the 5th day	382 patients (until the 3rd day all cultures under 20% O_2_): 189 patients for culture under 5% O_2_ (after the 3rd day). 193 patients for culture under 20% O_2_ (after the 3rd day).	- No statistically significant difference in the embryo quality, the implantation or the pregnancy rate was observed in the 5th day. - Similar implantation rates (44.06% under the 5% O_2_ vs. 44.16% under 20% O_2_), positive pregnancy tests (71.27% under the 5% O2 vs. 78.72% under 20% O_2_) and clinical pregnancies (58.56% under the 5% O2 vs. 64.36% under 20% O_2_). - The decrease in the oxygen levels has not proven to be beneficial, presumably due to the damage already caused during the first days of culture under 20% O_2_.	IVF
Kovacic et al. (2010) [62]	Prospective randomized study	Impact of embryo culture under different oxygen levels (6% CO_2_/5% O_2_/89% N_2_ or 6% CO_2_/20% O_2_) on the ICSI outcome	647 patients: 326 patients for culture under 5% O_2._ 321 patients for culture under 20% O_2._	- Despite the fact that 5% O_2_ levels led to a greater number of better-quality embryos on the 2nd day and optimal blastocysts, implantation rates (28.8% under 5% O_2_ and 25.2% under 20% O_2_) and rates of ongoing pregnancy (31.6% under 5% O_2_ and 27.1% under 20% O_2_) were similar in both groups. - The low oxygen levels resulted in higher pregnancy rates in general (38.8% under 5% O_2_ and 28.3% under 20% O_2_) and in the subgroup of poor responders (23% under 5% O_2_ and 9.8% under 20% O_2_).	ICSI
Sepulveda et al. (2011) [59]	Prospective randomized study	Comparison between embryo culture under 5% O_2_ or 20% O_2_	100 oocyte donation receivers randomized for culture under 5% O_2_ or 20% O_2_	- Fertilization rates were assessed at 72.4% under 5% O_2_ and 71.7% under 20% O_2_. - There was no significant difference in pregnancy rates (55.3% under 5% O_2_ and 54.6% under 20% O_2_). An increase in multiple pregnancies was observed (46.2% under 5% O_2_ and 29.2% under 20% O_2_). - Implantation rates emerged higher under low oxygen levels (41.8% under 5% O_2_ and 36.8% under 20% O_2_). - Cryopreservation was possible for 30.8% under 5% O_2_ and 19.2% under 20% O_2_. This difference was statistically significant. - Another statistically significant difference was observed in embryo quality (55.4% under 5% O_2_ and 41.8% under 20% O_2_).	IVF (not directly mentioned)
Sobrinho et al. (2011) [70]	Meta-analysis	Effect of low oxygen levels on fertilization, implantation and pregnancy rates	7 included studies: Dumoulin et al.(1999) [51], Bahceci et al. (2005) [61], Kea et al. (2007) [53], Kovacic et al. (2008) [55], Ciray et al. (2009) [64], Meintjes et al. (2009) [54] and Kovacic et al. (2010) [62].	- Fertilization rates, implantation rates and ongoing pregnancy rates were not significantly different between the 2 groups (5% O_2_ and 20% O_2_). - Implantation and ongoing pregnancy rates were similar in studies involving embryo transfer on the 2nd or the 3rd day. - 5% O_2_ levels emerged beneficial for embryo transfers on the 5th and 6th day, as confirmed by the statistically significant increase in implantation rates. - More randomized studies are advisable to clarify the effect of low oxygen levels in IVF.	IVF/ICSI
Bontekoe et al. (2012) [71]	Review with quantitative synthesis	Assessment of 5% O_2_ levels on IVF- and ICSI-related parameters	1382 patients from 4 included studies: Kovacic et al. (2008) [55], Meintjes et al. (2009) [54], Sepulveda et al. (2011) [59] and Waldestrom et al. (2011) [60].	- Embryo culture under 5% O_2_ is beneficial for IVF/ICSI outcomes. - Living birth rates, clinical pregnancy rates and ongoing pregnancies were positively affected. The estimated increase in success levels ranges from 32% to 43%. - An increase in multiple pregnancies, miscarriages and congenital deformities has not been observed.	IVF/ICSI
de los Santos MJ et al. (2013) [52]	Prospective randomized study	Evaluation of oxygen levels (5.5% CO_2_/6% O_2_/88.5% N_2_ or 5.5% CO_2_/20% O_2_) on ongoing pregnancies from oocyte donation cycles	564 cycles under 6% O_2._ 561 cycles under 20% O_2._	- Ongoing pregnancy rates were assessed at 41.3% under 6% O_2_ and at 40.8% under 20% O_2_. - Blastomere number and optimal embryo quality rate were statistically significantly higher at 6% O_2_. - In embryo transfers on the 3rd day, 6% O_2_ improved only embryo quality, without improving ongoing pregnancy rates.	IVF/ICSI
Kasterstein et al. (2013) [63]	Prospective randomized study	Evaluation of different oxygen levels (5% O_2_ or 20% O_2_) on embryo development and clinical outcome (in cycles with more than 8 oocytes collected)	258 patients. 3638 mature oocytes retrieved. 1833 incubated under 5% O_2._ 1805 sibling oocytes incubated under 20% O_2._	- Fertilization rates were similar in both groups. - The 5% oxygen levels resulted in statistically significantly increased blastomeres and optimal embryo quality. - More embryos were eligible for transfer in the low-oxygen group (31.6% under 5% O_2_ vs. 23.1% under 20% O_2_). - A statistically significant increase was observed in implantation and pregnancy rates in the low-oxygen group. - Living birth rates per transfer were also statistically significantly increased (34.2% under 5% O_2_ vs. 15.8% under 20% O_2_).	ICSI
Paternot et al. (2013) [65]	2 randomized controlled trials	Assessment of embryo incubation in a mini-incubator or a conventional incubator and effect of different oxygen levels (5% O_2_ and 20% O_2_) on embryo quality	395 embryos in each group	- Embryo cultures in a mini-incubator were similar to the embryo cultures in a conventional incubator on the first 3 days of embryo development. - Low oxygen concentrations did not affect embryo quality substantially.	IVF/ICSI
Peng ZF et al. (2015) [57]	Randomized study	Evaluation of different oxygen levels on fertilization rates, implantation rates, pregnancy rates, multiple pregnancies and miscarriages	3484 IVF and ICSI cycles: 1131 cycles cultured under 5% CO_2_ and 20% O_2._ 1258 cycles cultured at first under 5% CO_2_ and 20% O_2_ and after the 2nd day under 5% O_2_/5% CO_2_/90% N_2_ until the 3rd day. 1095 cycles cultured under 5% O_2_/5% CO_2_/90% N_2._	- Increased fertilization and implantation rates were statistically significant for culture under 5% O_2_/5% CO_2_/90% N2 in comparison to the other 2 groups. These data concern IVF embryos. - Embryos from IVF cultured under 20% O_2_ showed statistically significantly better fertilization rates, implantation rates and quality in comparison to the group with the oxygen levels’ shift. - Embryos from ICSI cultured under 20% O_2_ showed statistically significantly lower cleavage rates than those from the group with the oxygen levels’ shift. - These results lead to the conclusion that embryos are more sensitive to the oxygen levels’ shifts than to oxygen levels per se.	IVF/ICSI
Nastri et al. (2016) [72]	Systematic review and meta-analysis	Evaluation of different oxygen levels on embryo cultures	21 included studies	- Low-quality evidence suggested that low (5–6%) oxygen levels were beneficial for living birth and clinical pregnancy rate. - Low quality evidence showed no difference in fertilization and cleavage rate between low (5–6%) and high (20%) oxygen levels. - Low-quality evidence suggested that low (5–6%) oxygen levels resulted in better embryo development at the cleavage stage.	IVF/ICSI
Van Montfoort et al. (2020) [58]	Cohort study	Assessment of different oxygen levels’ potential involvement in embryo utilization, IVF success rates and birthweight	871 patients. 195 cycles in the group under 5% O_2_ (1627 oocytes). 676 cycles in the group under 20% O_2_ (5448 oocytes).	- A statistically significantly increased number of quality embryos has been observed under 5% O_2_ (45.8% under 5% O_2_ and 30.9% under 20% O_2_). - This increased number of quality embryos was not correlated to an increased living birth rate, but allowed embryos of better quality to be cryopreserved. - No correlation between oxygen levels and birthweight could be observed.	IVF or IVF/ICSI
Ruíz et al. (2020) [66]	Prospective randomized controlled trial	Assessment of the embryo culture under low oxygen levels (5% O_2_) in benchtop incubator on embryo-related parameters	148 patients: 73 patients in the control group culture under 20% O_2_ and in large box-incubators. 75 patients in the study group (culture under 5% O_2_ and in benchtop incubators).	- Implantation rate and live birth delivery rate per embryo transfer were significantly higher in the study group compared to the conventional embryo culture group (culture under 20% O_2_ and in large box-incubators). - Also, the cumulative implantation rate and the cumulative live birth rate per embryo transfer emerged as significantly higher in the study group compared to the conventional embryo culture group. - Particular benefit was assessed for poor-quality embryos. - Fresh embryo transfers were correlated with higher blastocyst, implantation, live birth, cumulative implantation and cumulative live birth rate in comparison to the frozen embryo transfers assessed.	IVF
Gelo et al. (2019) [67]	Prospective randomized controlled trial	Evaluation of embryo culture under 5% O_2_ in a benchtop incubator or 20% O_2_ in a classic incubator on embryo-related and clinical parameters	393 patients: 198 patients in the 5% O_2_ group. 195 patients in the 20% O_2_ group.	- Both for the live birth rates and for the fertilization rates, no statistically significant improvement could be assessed for the 5% O_2_ group. However, a statistically beneficial role of the embryo culture under 5% O_2_ was confirmed for the blastocyst number on the 5th day and for the fertilization rate. - Clinical pregnancy rates emerged as higher in the 5% O_2_ group, but significantly higher only on the 5th day. - Overall, benchtop incubators led to an increase in the number of blastocysts produced and, consequently, to higher pregnancy rates.	IVF/ICSI
Li et al. (2022) [68]	Randomized study	Assessment of ultra-low (2%) oxygen levels on embryo development and clinical parameters	2298 oocytes from 152 patients	- On the 3rd day, no statistically significant difference could be observed in good-quality and available embryos rates between the two groups (culture under 2% O_2_ and 5% O_2_). - The blastulation rate and the fraction of good-quality blastocysts on the 5th day and the total blastulation rate emerged as similar between the 2 groups. - Clinical and ongoing pregnancy rates were also similar in the first transfer between the 2 groups. - The application of embryo culture under 2% is not recommended based on these results.	IVF
Fawzy et al. (2017) [69]	Comparative study	Evaluation of low oxygen levels (3.5% O_2_) on embryo development and clinical parameters	6024 oocytes from 558 patients: 3290 oocytes from 293 patients in the 3.5% O_2_ group. 2734 oocytes from 265 patients in the 5% O_2_ group.	- Statistically significantly higher fertilization and cleavage emerged in the 3.5% O_2_ group compared to the 5% O_2_. - However, the compaction rate on the 3rd day and the number of formed, good-quality and cryopreserved blastocysts was assessed as significantly lower in the 3.5% O_2_ group. - Significantly lower biochemical pregnancy, clinical pregnancy and implantation rates were assessed for the 3.5% O_2_ group.	ICSI
Yang et al. (2016) [73]	Comparative study	Assessment of different oxygen levels (2% O_2_, 5% O_2_ and 20% O_2_) on embryo cultures until the blastocyst stage	155 embryos from 21 couples (120 finally included): 46 embryos in the 2% O_2_ group. 44 embryos in the 5% O_2_ group. 30 embryos in the 20% O_2_ group.	- The blastocyst formation rate was similar in the 3 groups assessed. - The apoptosis rate was significantly lower for blastocyst formation under 5% O_2_. - No statistically significant difference in the expression patterns of the genes assessed was found between the 2% O_2_ and the 5% O_2_ group. - Cryopreserved embryos from the 3rd day until the blastocyst stage will benefit from culture under 5% O_2_.	IVF
Kaser et al. (2018) [74]	Randomized controlled trial	Assessment of human preimplantation embryos’ culture under 2% O_2_ or 5% O_2_	203 zygotes: 102 zygotes for culture under 5% O_2_ for both periods (days 1 to 3 and days 3 to 5). 101 zygotes for culture under 5% O_2_ for days 1 to 3 and under 2% O_2_ for days 3 to 5.	- Embryos cultured under 2% O_2_ were less likely to arrest in cleavage stage on the 5th day. - A higher blastulation rate was assessed for embryos cultured under 2% O_2_. - Statistically significantly fewer cells were found in blastocysts of embryos cultures under 2% O_2_. - Metabolic changes, including amino acids’ capacity and metabolites’ status against oxidative stress, were also found. An overexpression of MUC1 was assessed in the 2% O_2_ without clinical significance.	IVF/ICSI
De Munck et al. (2019) [75]	2 prospective randomized controlled trial	Evaluation of the shift in oxygen concentration from 5% to 2% after the 3rd day on blastocyst parameters	1811 embryos (direct exposure). 405 embryos for culture under 2% O_2._ 406 embryos for culture under 5% O_2._ 1144 embryos (gradual exposure). 572 embryos for culture under 2% O_2._ 572 embryos for culture under 5% O_2._	- No statistically significant differences could be assessed in the blastulation rate, the utilization rate and the number of good-quality blastocysts.	IVF/ICSI
Brouillet et al. (2021) [76]	Monocentric retrospective observational study	Evaluation of oxygen levels’ shift from 5% to 2% on embryo-related and clinical parameters	120 couples. (1st IVF cycle with embryo culture under 5% O_2_ and 2nd IVF cycle with embryo culture under 5% O_2_ for the first 3 days and then under 2% O_2_ for days 3 to 5/6.)	- Usable blastocyst rate and the cumulative birth rate emerged as statistically significantly increased in the 2nd IVF cycle. - The expression pattern, according to the analysis of 707 RNAs, was significantly different and included genes related to many aspects of embryo development and beyond. - The oxygen levels’ shift may improve the effectiveness of IVF.	IVF
Li et al. (2022) [77]	Comparative study	Evaluation of oxygen levels’ shift from 5% to 2% in embryo-related and clinical parameters.	510 embryos from 188 patients: 296 embryos from 106 patients for culture under 5% O_2_ after the 3rd day until the 5th or the 6th day. 214 embryos from 82 patients for culture under 2% O_2_ after the 3rd day until the 5th or the 6th day.	- Blastulation rate and high-quality blastulation rate emerged as similar for high-quality embryos. - For low-quality embryos, culture under 2% O_2_ after the 3rd day improved the blastulation rate in a statistically significant way, without any significant improvement in the high-quality blastulation rate. - Prolonged culture until the 7th day led to a statistically significant increase in the blastulation rate for the low-quality embryos, cultured under 2% O_2._	IVF/ICSI
Patel et al. (2023) [78]	Retrospective cross-sectional study	Assessment of low (5% O_2_) and ultra-low (2% O_2_) oxygen levels on embryo-related and clinical parameters	382 patients; 206 embryos for culture under 2% O_2._ 176 embryos for culture under 5% O_2._	- Implantation and pregnancy rates by the oxygen concentration the two groups. - During frozen embryo transfer, the abortion rate was statistically significantly higher in the group of embryos cultured under 5% O_2_. - Despite the higher number of good-quality embryos observed in the 5% O_2_ group, clinical outcomes did not improve substantially. - Embryos transferred fresh showed a statistically significantly improved implantation rate.	IVF
Herbemont et al. (2021) [79]	Randomized controlled trial	Evaluation of the oxygen levels’ importance according to the embryo’s developmental stage	773 IVF/ICSI cycles: 265 cycles in culture under 20% O_2_ and after the 2nd day, culture of the available good-quality embryos in 88 cycles under 20% O_2._ 508 cycles in culture under 5% O_2_, after the 2nd day either culture until the 6th day for 195 cycles or shift of the oxygen levels to 20% O_2_ until the 6th day for 94 cycles.	- The number of early embryos in the cleavage state and the quality features for embryos on the 2nd day were statistically significantly higher in the group with the 508 cycles performed. A proper model also confirmed the significance of the oxygen levels in that correlation. - The number and the rate of top-quality blastocysts on the 5th day emerged as statistically significantly lower in the group with the 88 cycles performed. The groups with the 195 and 88 cycles performed exhibited similar results. The first outcome was confirmed for the 6th day as well. - After the transfer of the blastocysts, no important differences were observed regarding the clinical parameters. - Overall, the researchers propose the culture of the embryos at an early stage under low oxygen levels, as the oxidative stress acts presumably prior to the genome activation of the embryo.	IVF/ICSI

## 6. Conclusions

The cultures under low or high oxygen levels have been extensively investigated either in embryos of various mammal species or human embryos. The hasty conclusion from the animal studies is that the culture in low oxygen levels, instead of the culture in atmospheric oxygen levels, offers advantages in embryo development. However, the optimal low oxygen levels vary by species. As for human embryos, most of the studies compared 5% to 20% oxygen levels. Although the quality of the evidence is not good, as the meta-analysis of Nastri et al. [72] indicates, all the relevant studies, with the exception of one [64], showed that the culture under low oxygen levels is, to some extent, beneficial for human embryos. Nevertheless, no study to date could confirm any benefit for embryos, implantation or pregnancy rates related to the use of atmospheric oxygen levels in the human embryo culture in comparison to the culture under 5–6% oxygen levels. The biphasic oxygen concentration strategy (5% in the first three days and 2% afterwards) appears as an interesting and promising method, though additional studies are needed in order to confirm its value.

In conclusion, the currently available evidence does not support the culture of human embryos under atmospheric conditions. The higher cost related to cultures under 5% or 2% O_2_ cannot be considered as a reason for not adopting the culture under low oxygen tension.

## Data Availability

No new data were created.
